# Diagnosis Test Meta-Analysis for Apolipoprotein E in Alzheimer's Disease

**DOI:** 10.1155/2020/6486031

**Published:** 2020-10-09

**Authors:** Xuan Xiong, Hongtao Xiao, Yuan Zhang, Dongke Yu, Junlan Chuan, Lei Zhong, Qinan Yin, Rongsheng Tong

**Affiliations:** ^1^Department of Pharmacy, Sichuan Academy of Medical Sciences & Sichuan Provincial People's Hospital, Chengdu610072, Sichuan Province, China; ^2^Personalized Drug Therapy Key Laboratory of Sichuan Province, School of Medicine, University of Electronic Science and Technology of China, Chengdu 610072, Sichuan Province, China; ^3^Department of Pharmacy, Sichuan Cancer Hospital & Institute, The Affiliated Cancer Hospital, School of medicine, University of Electronic Science and Technology of China, Chengdu 610072, Sichuan Province, China

## Abstract

**Objective:**

To evaluate the diagnostic value of apolipoprotein E (*APOE*) gene in Alzheimer's disease (AD).

**Methods:**

Databases including PubMed, EMBASE, Google Scholar, Wanfang Med online, China National Knowledge Infrastructure (CNKI), and China Biomedical Literature Database (CBM) were searched for literatures in English or Chinese. No limitations on the date. The sensitivity, specificity, likelihood ratio, and diagnostic odds ratio were pooled for meta-analysis. The symmetric receiver operator characteristic curve (SROC) and Fagan's Nomogram were drawn, and metaregression and subgroup analysis were used to explore the source of heterogeneity.

**Results:**

A total of 13 studies, including 2662 cases and 8843 controls, were analyzed. The combined sensitivity (SEN) was 0.62 (95% CI (0.58-0.66)), specificity (SPE) was 0.84 (95% CI (0.81-0.86)), the positive likelihood ratio was 3.8 (95% CI (3.3-4.3)), and the negative likelihood ratio was 0.45 (95% CI (0.41-0.49)). The area under the ROC curve was 0.80, and the diagnostic ratio (DOR) was 8. Neither publication bias was detected in Deeks' funnel plot, nor threshold effect was shown in the SROC. Metaregression analysis showed that the diagnostic methods, experimental design, and sample size contributed to the heterogeneity in SEN, while the diagnostic methods, experimental design, blind evaluation on test results, and sample size contributed to the heterogeneity in SPE. When the pretest probability was set as 50%, the posterior probability in Fagan's Nomogram was 79%, the positive likelihood ratio (LRP) was 5, and the negative likelihood ratio (LRN) was 0.42.

**Conclusions:**

AD could neither be confirmed nor excluded by the *APOE* genotype test. The sensitivity and specificity of the *APOE* gene test were relatively low in the diagnosis of AD. The diagnostic value of *APOE ε*4 gene in AD was moderate; it might play an important role in the prevention of AD.

## 1. Introduction

Alzheimer's disease (AD), known as primary Alzheimer's disease, is a progressive neurodegenerative disease with occult onset, accounting for 50%-60% of all types of dementia [1]. An epidemiological survey in four major cities including Beijing, Shanghai, Chengdu, and Xi'an showed that the prevalence of AD in the elderly (>65 years old) was 3.4% in males, 7.7% in females, and 5.9% in total [2]. The main clinical manifestations of AD are memory disorder, cognitive dysfunction, language disorder, and personality and behavior changes, which seriously affect the quality of life of patients and their families with burden of care. For the aging China, the number of AD patients is rising to the peak year by year, while the treatment rate is very low [3]. Therefore, the work in AD prevention and treatment has been one of the key tasks for the government and the medical community.

At present, the etiology of AD has not been fully understood. Amyloid cascade hypothesis is widely accepted. This theory proposes that amyloid-beta (*β*-amyloid protein (A*β*)) is the key point [4]. The accumulation of A*β* sets off a series of events which has toxic effects on peripheral synapses and neurons that results in the death of brain cells and, eventually, Alzheimer's disease [5]. Apolipoprotein E (ApoE) is closely related to A*β* metabolism in the central nervous system, and the subtype can significantly affect the age spots formed by A*β* deposition. The best characterized *APOE*-associated risk for AD is due to the *ε*4 allele, which is an important risk factor. And *ε*2 has a protective effect on Alzheimer's disease, commonly known as “longevity factor” [6]. At present, *APOE* genotype has been used as an assistant diagnosis of AD in clinical practice, but its diagnostic value has not been reported in detail. In this research, the diagnostic value of *APOE ε*4 on AD will be studied by meta-analysis; a quantitative reference for clinical practice will also be provided.

## 2. Materials and Methods

### 2.1. Inclusion and Exclusion Criteria

#### 2.1.1. Inclusion Criteria

1. The research content includes the application of *APOE* genotype detection in patients with Alzheimer's disease
2. The mental detection scale was recommended by the research group for imaging, histopathology and/or national guidelines, such as the World Health Organization's International Classification of Diseases, 10th Edition (ICD-10), the American Manual of The Diagnostic and Statistical Manual of Mental Disorders (DSM), and the National Neurolinguistic Disorder of the United States. According to the diagnostic criteria of National Institute of Neurological and Communicative Disorders and Stroke-Alzheimer's Disease and Related Disorders Association (NINCDS-ADRDA), and Classification and Diagnostic Criteria of Mental Disorders in China(CCMD), the patients with Alzheimer's disease (including mild, moderate, and severe ones) were diagnosed
3. The control group was the elderly without neurodegenerative diseases who had no significant difference in age and gender
4. Each *APOE* genotype was reported by exact cases: (*ε*2/*ε*2, *ε*2/*ε*3, *ε*3/*ε*3, *ε*2/*ε*4, *ε*3/*ε*4, and *ε*4/*ε*4, where *ε*2/*ε*2, *ε*2/*ε*3, *ε*3/*ε*3, and *ε*2/*ε*4 were considered negative, and *ε*3/*ε*4 and *ε*4/*ε*4 were considered positive)

#### 2.1.2. Exclusion Criteria

The exclusion criteria were as follows: (1) review, case report, and animal experiment; (2) repeated study; (3) study with incomplete data, unable to calculate the four grid data (i.e., the numbers of true positive, false positive, false negative, and true negative cases); and (4) not Chinese or English.

### 2.2. Search Strategy and Article Selection

The following search databases were used: PubMed, EMBASE, Google Scholar, Wanfang Med online, CNKI, and CBM. The searching time was up to December 2017. The search term was “APOE and Alzheimer's disease.”

### 2.3. Data Extraction and Quality Assessment

Two evaluators independently screened and extracted the literature according to the acceptance and discharge criteria and cross-checked them. The content of data extraction includes article title, name of the first author, publication period, research country and race, diagnostic standard, total number of samples, experimental design, and four grid table data.

According to the quality assessment of diagnostic accuracy studies-2 (QUADAS-2), 14 items were evaluated according to the three criteria of “yes,” “no,” or “unclear”: “1” point for “yes,” “-1” point for “no,” and 0 point for unclear.

### 2.4. Statistical Analysis

The Midas command package of Stata 12.0 software was used to realize the meta-analysis of diagnostic accuracy data. Midas command uses the bivariate model to calculate the combined sensitivity (SEN), specificity (SPE), positive likelihood ratio (PLR), negative likelihood ratio (NLR), and diagnostic ratio (DOR) and draw the SROC curve to estimate the total diagnostic accuracy of the test. Fagan diagram is used to test the posttest probability. *Q*-test and *I*^2^ were used to evaluate the degree of heterogeneity between studies. If *I*^2^ was more than 50%, heterogeneity was considered.

If there is heterogeneity caused by nonthreshold effect, univariate regression analysis and subgroup analysis are used to explore the source of heterogeneity. In this study, the source of heterogeneity is mainly considered from the following aspects: diagnostic criteria, test design, blind evaluation of test results, and sample size. Publication bias was detected by Deeks' funnel chart.

## 3. Results

### 3.1. Flow Chart and Study Quality

**Figure 1:**
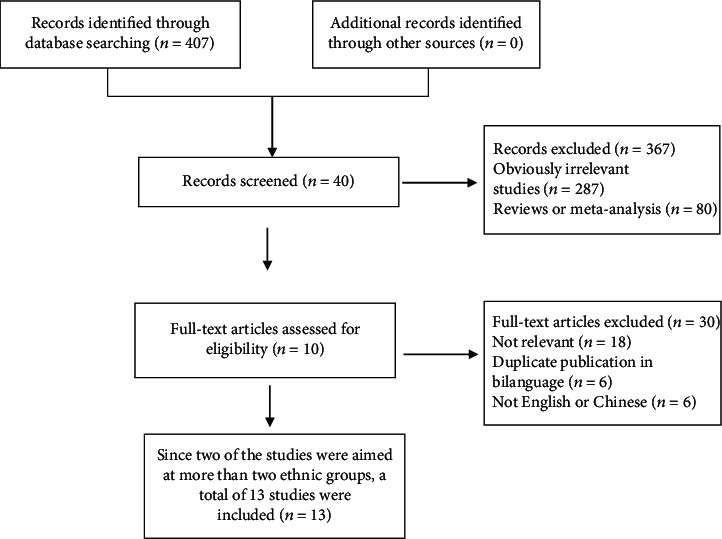
Literature screening process of the meta-analysis.

407 papers (including documents, reviews, animal experiments, case reports, and repeated studies) were retrieved from each database; 367 of the non-Chinese or English papers were kicked out after abstract reading. The full text of remaining 40 were extracted. 30 studies with incomplete data in four tables were removed after reading the full text. The remaining 10 papers were extracted from the corresponding data according to the data extraction requirements. Because Ganguli et al. reported Indian data of two regions and Tang et al. reported data of black, white, and Hispanic Americans, 13 data were included in this study. The literature screening process can be seen in Figure 1. The basic characteristics and QUADAS-2 scores of each study included can be seen in Table 1.

### 3.2. Statistical Analysis

#### 3.2.1. Consolidation Statistics

**Figure 2:**
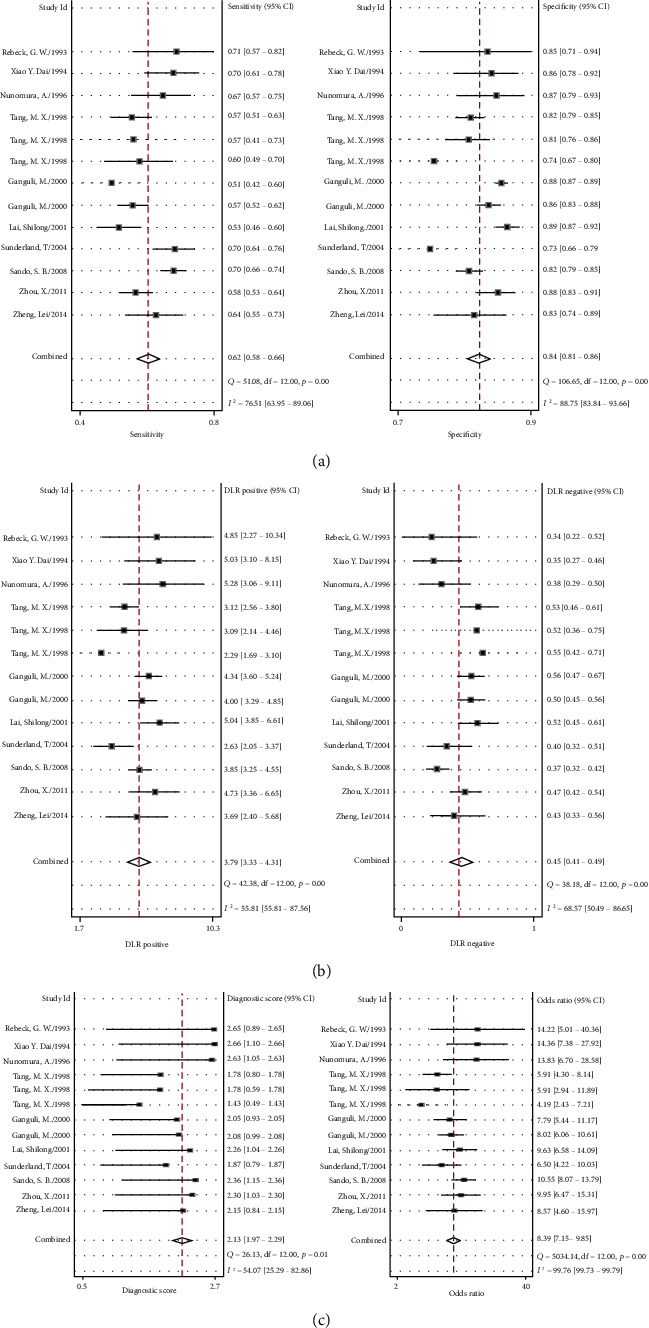
(a) Forest plot of sensitivity and specificity of *APOE* genotype in the diagnosis of Alzheimer's disease. (b) Forest plot of DLR positive and negative of Alzheimer's disease. (c) Forest map of the diagnostic odds ratio of *APOE* genotype in Alzheimer's disease.

The meta-analysis of diagnostic accuracy data was realized by using Stata Midas command. The combined sensitivity was 0.62 (95% CI (0.58, 0.66)), specificity was 0.84 (95% CI (0.81, 0.86)), positive likelihood ratio was 3.8 (95% CI (3.3, 4.3)), negative likelihood ratio was 0.45 (95% CI (0.41, 0.49)), area under ROC curve was 0.80, and diagnostic ratio (DOR) was 8, which indicated the *APOE ε*4 has a medium value in the diagnosis of AD. The test of heterogeneity is *I*^2^ = 97, highly heterogeneous. The details of combined sensitivity and specificity forest can be seen in Figure 2(a), the combined likelihood ratio forest in Figure 2(b), and the combined diagnosis ratio forest in Figure 2(c).

#### 3.2.2. Publication Bias

**Figure 3:**
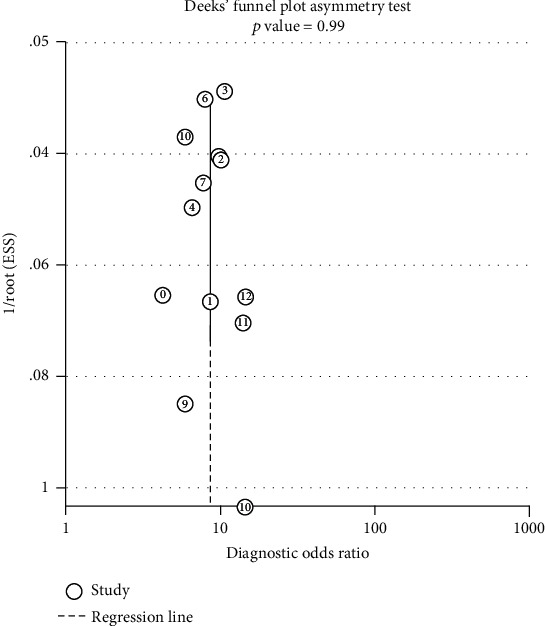
Deeks' funnel plot asymmetry test.

Midas used linear regression to test funnel asymmetry to evaluate publication bias. The digital results showed that the linear regression test *p* was 0.991, indicating that there was no asymmetry in funnel diagram (*p* < 0.01; the difference was statistically significant). The possibility of publishing bias was very small since the angle between the regression line and the horizontal axis (DOR axis) was very close to 90°; the details can be seen in Figure 3.

#### 3.2.3. Threshold Effect

**Figure 4:**
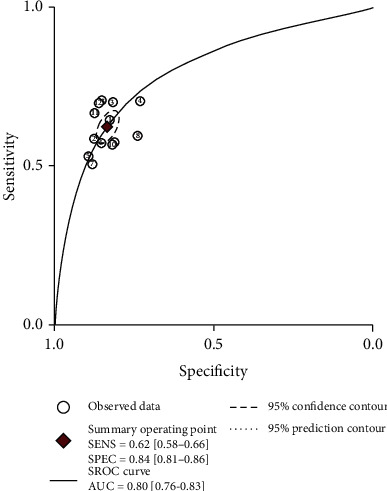
Summary receiver operating characteristic.

The threshold effect can be judged according to the SROC curve plane test. Since there was no typical “shoulder arm”, it can be concluded that there might be no threshold effect. As the correlation coefficient of sensitivity logarithm and the *p* value of 1-specificity logarithm were -0.78 and *p* = 0.61, respectively, it can be inferred that the threshold effect was not significant. However, since the Cochran's *Q* value was 59.49 and the *p* value was less than 0.05, which indicated the heterogeneity was caused by the non threshold effect, a moderate diagnostic value could be concluded by the value of the area under the SROC curve (AUC), which was 0.80 (95% CI: 0.76-0.83). The details of the SROC curve are shown in Figure 4.

#### 3.2.4. Metaregression and Subgroup Analysis

**Figure 5:**
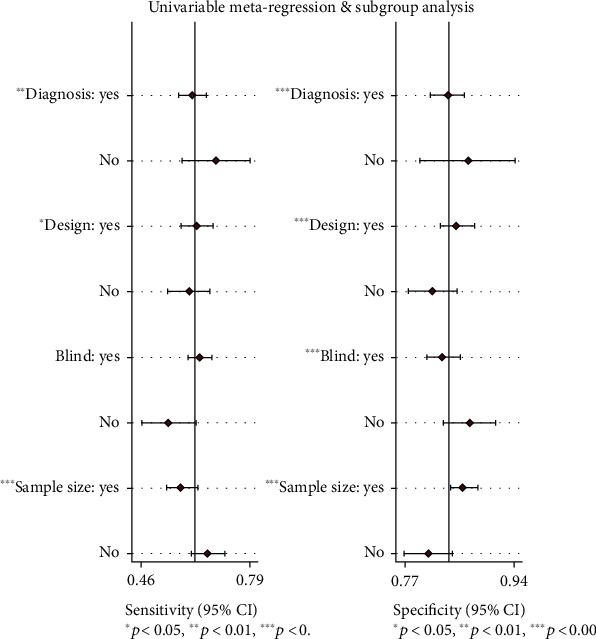
Single factor metaregression and subgroup analysis. Diagnosis—the scale: yes; the nonscale: no. Design—case control study: yes; a cross-sectional study: no. Blind—blind study: yes; nonblinded study: no. Sample size—over 300: yes; below 300: no.

In this study, the factors brought to heterogeneity caused by a nonthreshold effect included diagnostic criteria (“1” for scale evaluation, 0 for nonscale evaluation, such as image or biopsy), trial design (1 for case-control, 0 for cross-sectional), and whether to evaluate the test results by blind method (1 for nonblind method, 0 for blind method) and sample size (0 for more than 300 people and less than 1300 people). As shown in Table 2, through the metaregression analysis of the above factors, it was found that although the sources of heterogeneity of SEN were statistically related to the diagnosis method, test design, and sample size, while the sources of heterogeneity of SPE were related to the diagnosis method, test design, blind evaluation of test results, and sample size, there was no significant difference in clinical significance. The details can be seen in Figure 5.

#### 3.2.5. Pretest Probability, Likelihood Ratio, and Posttest Probability

**Figure 6:**
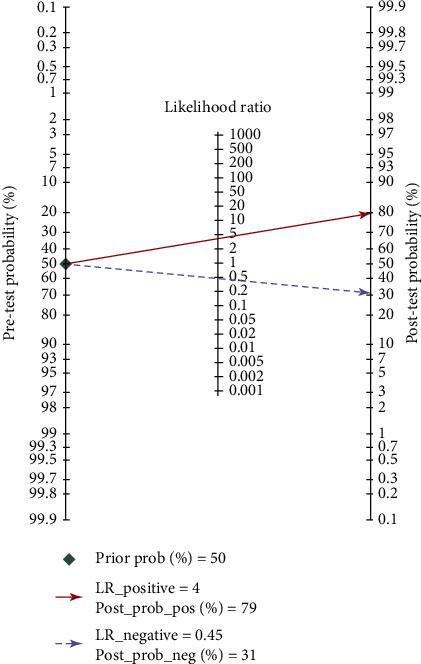
Fagan diagram of *APOE* genotype in the diagnosis of Alzheimer's disease.

The Fagan graph was plotted to show the relationship among the prior probability, the likelihood ratio, and the posterior probability. The pretest probability was 50%, the *APOE* test results were high-risk (i.e. *ε*3/*ε*4 and *ε*4/*ε*4), and the probability of Alzheimer's disease was 79%. In addition, positive likelihood ratio (LRP) was <10 (LRP = 5) and negative likelihood ratio (LRN) was >0.1 (LRN = 0.42), indicating that the diagnosis can neither be confirmed nor excluded. Their diagnostic value of *APOE ε*4 in AD was limited. The details can be seen in Figure 6.

## 4. Discussions

AD is the primary cause of dementia. It is one of the major diseases that cause death and disability in the elderly, and it affects the quality of life for patients and their families [17]. So far, the pathogenesis of AD is not clear. For the treatment of AD, whether it is drug therapy, physical therapy, or psychological therapy, it can only delay the development of the disease, it can not interrupt the progress of the course of AD, nor can it be completely cured [18]. Therefore, early diagnosis and timely intervention are the only effective measures to delay the progress of this disease [19]5. At present, there are three sets of international diagnostic standards for AD, namely, the National Institute of Neurological and Communicative Disorders and Stroke-Alzheimer's Disease and Related Disorders Association (NINCDS-ADRDA) standards, International Classification of Diseases 10th Edition (ICD-10) of the World Health Organization, and the 5th Edition of Diagnostic and Statistical Manual of Mental Disorders (DSM-IV-TR) [20–21]. None of the three standards mentioned the application of biomarkers. With the development of AD research, new AD diagnostic standards come up, such as International Working Group-2 (IWG-2) criteria issued by the International Working Group and Alzheimer's Association of the National Institute of Aging and National Institute on Aging and Alzheimer's Association (NIA-AA) issued by the USA National Institute of Aging [22–23]. The new standards include biomarkers such as A*β* 1-42 combined with total tau or phosphorylated tau protein level in cerebrospinal fluid (CFS), amyloid PET imaging, autosomal dominant mutation (e.g., PSEN1, PSEN2, and APP mutations) in the diagnosis and identification of AD [23]. However, the main disadvantage of the three sets of scales is that they can only be used when the patients have dementia and/or their daily ability is affected; the best opportunity for intervention is missed. Because the biomarkers in the CSF need operation, the patients often have concerns about this, so the application is limited. Although people with PSEN1, PSEN2, and APP mutations will get sick sooner or later, such patients only account for about 1% and 15% of all Alzheimer's patients [24]. Therefore, for the more common sporadic Alzheimer's patients, it is of great significance to carry out prevention work.

In this study, 2662 cases and 8843 controls were analyzed by meta-analysis. The results showed that the sensitivity of *APOE* genotype in the diagnosis of Alzheimer's disease was 0.62 (0.58-0.66), the specificity was 0.84 (0.81-0.86), and the area under the SROC curve was 0.80 (0.76-0.83). It is suggested that *APOE* genotype may be useful in the diagnosis of Alzheimer's disease. If the diagnosis of AD is combined with the *APOE ε*4 gene test and psychiatric scale, assuming that the physician estimates the probability of AD based on the patient's history and physical signs and the results of the scale test is 50% (the diagnostic accuracy of NINCDS-ADRDA is reported to be 65%-96% [25]), then, the posterior probability is 79%. This result is of great significance for the diagnosis and prevention of early AD, but it has limited value in distinguishing other types of dementia: some scholars reported in the early research that the specificity of *APOE ε*4 detection in distinguishing ad-induced dementia and other types of dementia is only 23%-88% [26], so it is necessary to combine other detection methods to exclude other factors that caused dementia.

However, there are still limitations in this study:

1. The number of included studies is less, and the number of covariates is more in regression analysis, which may lead to the probability of multiple comparisons making class I errors
2. Although a few of the included studies reported the race of the subjects, most of the studies did not mention it, so it is impossible to analyze the influence of race factors on the value of *APOE* in AD diagnosis
3. Most of the patients included in the study did not mention the degree of AD. In patients with mild or suspected AD or moderate or severe AD, the sensitivity and specificity of calculated *APOE* may be different. Due to the insufficient data, this study does not discuss the degree of AD hierarchically
4. The diagnostic criteria adopted are not uniform enough. Most of the studies have adopted the international recommended ICD or DSM scale, but some of the studies have adopted other mental scales or scales plus other diagnostic experiments, which is one of the reasons for the heterogeneity between the studies

## 5. Conclusions

AD could neither be confirmed nor be excluded by the *APOE* gene test. The sensitivity and specificity of *APOE* gene test were relatively low in the diagnosis of AD. The diagnostic value of *APOE ε*4 gene in AD was moderate; it might play an important role in the prevention of AD.

## Figures and Tables

**Table 1:** Main characteristics of the studies included in the meta-analysis.

Study	Year	Country	Ethnicity	The way for diagnosis	Number of true positive cases	Number of false positive cases	False negative cases	True negative cases	The design of experiment	The blind method	QUADAS-2 score
Zheng [7]	2014	China	Asian	CCMD-3	76	19	42	90	Case-control	NM	10
Zhou [8]	2011	China	Asian	CMMSE	209	31	149	220	Case-control	NM	9
Sando [9]	2008	Norway	Caucasian	NINCDS-ADRDA	376	125	160	561	Case-control	NM	10
Sunderland [10]	2004	USA	Whites	MMSE, CDR, GDS, DRS	150	52	63	142	Cross-sectional	NM	10
Lai [11]	2001	China	Asian	DSM-III-R, NINCDS-ADRDA	110	61	97	518	Cross-sectional	NM	10
Ganguli [12]	2000	USA	Monongahela Valley	NINCDS-ADRDA	247	107	184	639	Case-control	YES	11
Ganguli [12]	2000	USA	Ballabgarh	NINCDS-ADRDA	64	581	62	4386	Case-control	YES	11
Tang [13]	1998	USA	African Americans	CDR	53	45	36	128	Cross-sectional	NM	10
Tang [13]	1998	USA	Whites	CDR	23	49	17	214	Cross-sectional	NM	10
Tang [13]	1998	USA	Hispanics	CDR	145	115	110	516	Cross-sectional	NM	10
Nunomura [14]	1996	Japan	Asian	CT/MRI, ICD-10	72	12	36	83	Case-control	NM	9
Xiao [15]	1994	Japan	Asian	NINCDS-ADRDA	88	15	38	93	Case-control	NM	10
Rebeck [16]	1993	USA	Whites	Biopsy	39	6	16	35	Case-control	NM	8

CMMSE: Chinese Mini-Mental State Examination; CCMD-3: Chinese Classification and Diagnosis of Mental Diseases-3rd; NINCDS-ADRDA: National Institute of Neurological and Communicative Disorders and Stroke-Alzheimer's Disease and Related Disorders Association; ICD-10: International Classification of Diseases 10th Edition; MMSE: Mini-Mental State Examination; CDR: Clinical Dementia Rating; GDS: Global Deterioration Scale; DRS: Dementia Rating Scale; DSM-III-R: Diagnostic and Statistical Manual of Mental Disorders-III-R; CT: Computed Tomography; MRI: Magnetic Resonance Imaging; NM: not mentioned.

**Table 2:** Metaregression and subgroup analysis.

Variable	Category	Number of study	Sensitivity (95% CI)	*p*_1_	Specificity (95% CI)	*p*_2_
Diagnostic criteria	Checklist	11	0.62 (0.58-0.66)	0.01	0.83 (0.81-0.86)	≤0.001
	Non-checklist	2	0.69 (0.58-0.79)		0.87 (0.79-0.94)	
Experimental design	Case-control	8	0.63 (0.58-0.68)	0.04	0.85 (0.82-0.88)	≤0.001
	Cross-sectional	5	0.61 (0.54-0.67)		0.81 (0.77-0.85)	
Blind	No	11	0.64 (0.60-0.67)	0.68	0.83 (0.80-0.85)	≤0.001
	Yes	2	0.55 (0.46-0.63)		0.87 (0.83-0.91)	
Sample size	≥300	6	0.59 (0.54-0.63)	≤0.001	0.86 (0.84-0.88)	≤0.001
	<300	7	0.66 (0.62-0.71)		0.80 (0.77-0.84)	

## Data Availability

The data used to support the findings of this study are available from the corresponding author upon request.
